# Effect of Low-Dose Ionizing Radiation on the Expression of Mitochondria-Related Genes in Human Mesenchymal Stem Cells

**DOI:** 10.3390/ijms23010261

**Published:** 2021-12-27

**Authors:** Svetlana V. Kostyuk, Elena V. Proskurnina, Marina S. Konkova, Margarita S. Abramova, Andrey A. Kalianov, Elizaveta S. Ershova, Vera L. Izhevskaya, Sergey I. Kutsev, Natalia N. Veiko

**Affiliations:** Laboratory of Molecular Biology, Research Centre for Medical Genetics, 1 Moskvorechye St., 115522 Moscow, Russia; svet-vk@ya.ru (S.V.K.); mkonkova@gmail.com (M.S.K.); rimargarii@gmail.com (M.S.A.); googlbubu@gmail.com (A.A.K.); es-ershova@rambler.ru (E.S.E.); izhevskaya@med-gen.ru (V.L.I.); kutsev@mail.ru (S.I.K.); satelit32006@yandex.ru (N.N.V.)

**Keywords:** low-dose ionizing radiation, mitohormesis, cell-free DNA, human mesenchymal stem cells, mitochondria

## Abstract

The concept of hormesis describes a phenomenon of adaptive response to low-dose ionizing radiation (LDIR). Similarly, the concept of mitohormesis states that the adaptive program in mitochondria is activated in response to minor stress effects. The mechanisms of hormesis effects are not clear, but it is assumed that they can be mediated by reactive oxygen species. Here, we studied effects of LDIR on mitochondria in mesenchymal stem cells. We have found that X-ray radiation at a dose of 10 cGy as well as oxidized fragments of cell-free DNA (cfDNA) at a concentration of 50 ng/mL resulted in an increased expression of a large number of genes regulating the function of the mitochondrial respiratory chain complexes in human mesenchymal stem cells (MSC). Several genes remained upregulated within hours after the exposure. Both X-ray radiation and oxidized cfDNA resulted in upregulation of *FIS1* and *MFN1* genes, which regulated fusion and fission of mitochondria, within 3–24 h after the exposure. Three hours after the exposure, the number of copies of mitochondrial DNA in cells had increased. These findings support the hypothesis that assumes oxidized cell-free DNA as a mediator of MSC response to low doses of radiation.

## 1. Introduction

The effects of low-dose ionizing radiation (<0.5 Gy) (LDIR) on stem cells remain poorly understood, although many studies indicate a significant effect of LDIR on cells through oxidative stress [1,2,3]. While moderate doses (1 Gy) led to the induction of apoptosis in stem cells, low doses of 0.2–0.5 Gy did not induce a detectable change in apoptosis compared to non-irradiated cells [4,5]. Stem cells live in a human body for a long time that increases the probability of accumulating genotoxic damage as a result of exposure to external or internal damaging factors. Cells can restore DNA integrity, but in the case of extensive DNA damage, the cells accumulate irreversible disruptions, which can enhance apoptosis or lead to mutations and malignization [6]. Therefore, long-lived stem cells can be considered as a key object for studying the effect of low doses of ionizing radiation [7], which can be used for the prediction of the risk to human health [6,8]. However, such important biological features of stem cells as clonogenic potential, ageing and autophagy are poorly understood that necessitates a more thorough study of the effect of LDIR on stem cells [5]. However, because of different doses, exposure times, and various populations of stem cells obtained from different sources, it is difficult to make a general conclusion about these effects [5,9].

Mitochondria provide metabolic activity in cells providing them with energy through oxidative phosphorylation. The morphology of mitochondria varies in tissues and depends on the metabolic state of cells [10]. In addition to the different number of mitochondria in different tissues, mitochondria are affected by the synthesis/division mechanism, which is closely related to the proliferation and differentiation of stem cells. It was shown that the activation of mitochondrial fusion precedes the differentiation of stem cells [10].

Mitochondria are essential not only for many energetic and fundamental biological processes. Nowadays, mitochondria are considered as signaling hubs in a cell, capable of affecting the cell they are in and other cells through changes in the mitochondrial proteome [11]. The concept of mitohormesis is based on a fine balance, where low-level mitochondrial stress induces an adaptive response increasing health and life span [12]. Mitohormesis lies in the phenomenon that mitochondrial stress rapidly activates cytosolic signaling pathways that affect the expression of nuclear genes protecting against stress [13].

Ionizing radiation influences mitochondria in a variety of ways. Mitochondrial DNA (mtDNA) is significantly more susceptible to radiation than genomic DNA because it does not have the same repair mechanisms as nuclear DNA has. Moreover, mtDNA does not contain histones that leads to a decrease in resistance to damaging effects [14]. Radiation-induced mitochondrial damage can induce apoptosis, mitochondrial autophagy, or, in less severe cases, fusion. This mechanism provides complementarity between damaged mitochondria and maintaining their functionality. X-ray radiation at doses from 0.005 to 5.0 Gy leads to an increase in the mass of mitochondria by a factor of 1.5–3.8, which confirms the theory of an increase in mitochondrial fusion after irradiation [10,15]. In stem cells exposed to radiation, metabolic activity increases, which may induce mitochondrial fusion and, therefore, stimulate the stem cell differentiation [16].

At the moment when the research of the effects of LDIR had started, a paradoxical phenomenon was revealed. Low-dose radiation had stimulating effects on cell cultures and in vivo, prolonging the life span of experimental animals and increasing cell survival [17]. The stimulating phenomenon of low-dose radiation was called a hormesis. Within this concept, moderate stressors result in an adaptive response that strengthens the body’s defenses. According to the linear no-threshold (LNT) model, the effect of radiation is proportional to the radiation dose. Researchers mainly use the LNT model, although many facts contradict it [18]. Adaptation to low doses of radiation was an evolutionary process, since in the early periods of life were on the Earth with a higher radiation background than that existing today [19]. Despite numerous studies, substantial knowledge gaps exist in our understanding of the molecular mechanisms that govern biological responses and health outcomes upon exposure to low-dose radiation [20].

Here, we aimed to study the effects of low-dose ionizing radiation on the expression of genes that regulate the function of mitochondria in mesenchymal stem cells and to find possible links between the radiation hormesis and mitohormesis.

## 2. Results

### 2.1. Low-Dose Ionizing Radiation Induce a Short-Term Increase in Intracellular ROS in MSCs

**Figure 1:**
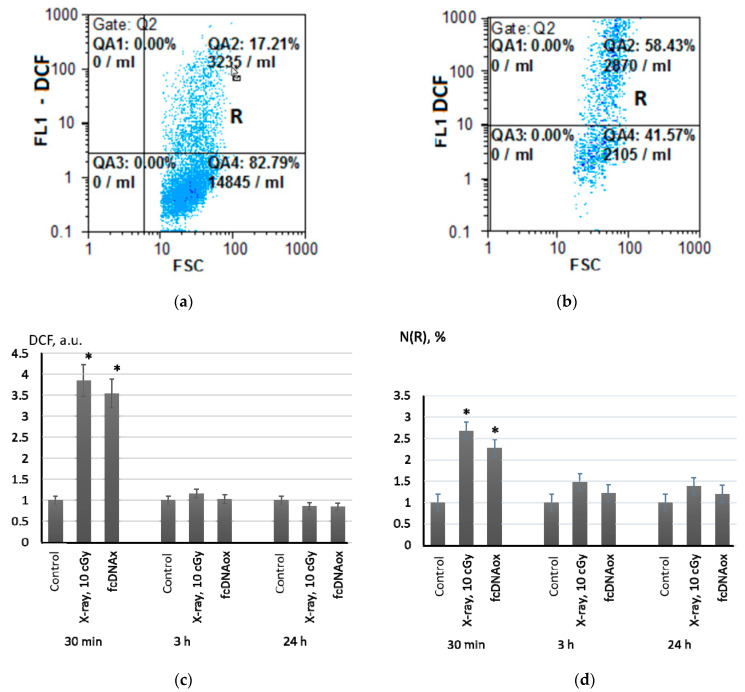
Intracellular ROS after X-ray radiation (10 cGy) and addition of the standard solution of cfDNAox (50 ng/mL) assessed by flow cytometry with a H2DCFH-DA dye; (**a**) FL1-DCF versus SSC for the control experiment; (**b**) FL1-DCF versus SSC for irradiated cells after 30 min; (**c**) histograms of DCF fluorescence measured by FACS; (**d**) histograms of R-fraction fluorescence in the MSCs. The histograms were built from three independent measurements on four cell cultures; mean values and standard deviations are given; (*) denotes a significant difference from control experiments, *p* < 0.001, nonparametric U-test.

The detection and quantitation of ROS in MSCs was carried out by flow cytometry with 2,7-dichlorodihydrofluorescein diacetate (H2DCFH-DA), which forms a fluorescent product (DCF) being oxidized by ROS (Figure 1a,b).

Radiation at a dose of 10 cGy led to increasing in intracellular ROS by a factor of 3.8 within 20 min with subsequent decreasing in ROS to control values after 3 h of irradiation (Figure 1c). A highly fluorescent R-fraction of the MSCs was detected in the cell population (Figure 1d). The amount of these cells and their fluorescence increased in 20–30 min after irradiation by a factor of 2.3–2.6. Three hours after the irradiation, the fluorescence of the R-fraction decreased, but remained higher than the control values by a factor of 1.4–1.6 even within 24 h (Figure 1d).

We have shown previously that X-ray radiation at a dose of 10 cGy resulted in formation of oxidized cell-free DNA (cfDNAox) originating from dead MSC cells [21,22]. We hypothesized that cfDNAox may serve as a mediator promoting ROS synthesis in MSCs. To exclude the influence of methylation and variations in the content of the sequences, we prepared standard oxidized DNA fragments. The oxidation level in these standard fragments was equal to the oxidation level in cfDNA isolated from the culture medium of the MSCs after irradiation.

In parallel with irradiation (10 cGy), we added oxidized cfDNA (50 ng/mL) to the MSC culture medium and analyzed intracellular ROS. As low-dose X-ray radiation did, the oxidized cfDNA induced short-term ROS synthesis in the MSCs. During the first 30 min, intracellular ROS increased sharply by a factor of 2.8–3.5 and fell to the control values within 3 h (Figure 1c). A half an hour after addition of standardized cfDNAox, the amount of high fluorescent R-fraction had increased by a factor of 1.8–2.4. Three hours later, the fluorescence of the R fraction had decreased to the control values (Figure 1d).

To sum, low-dose X-ray radiation, as well as oxidized cfDNA, lead to short-term intracellular oxidative stress. We have previously shown that an increase in intracellular ROS is a consequence of the activation of NADPH oxidases, one of which is NOX4 [21,22,23]. Another source of intracellular ROS is a mitochondrial respiratory chain. Therefore, we studied the mitochondrial potential in MSCs using a Mito-tracker Red 580 mitochondrial dye (TMRM Red).

### 2.2. Effects of Low-Dose Radiation and Oxidized cfDNA on Mitochondrial Potential

**Figure 2:**
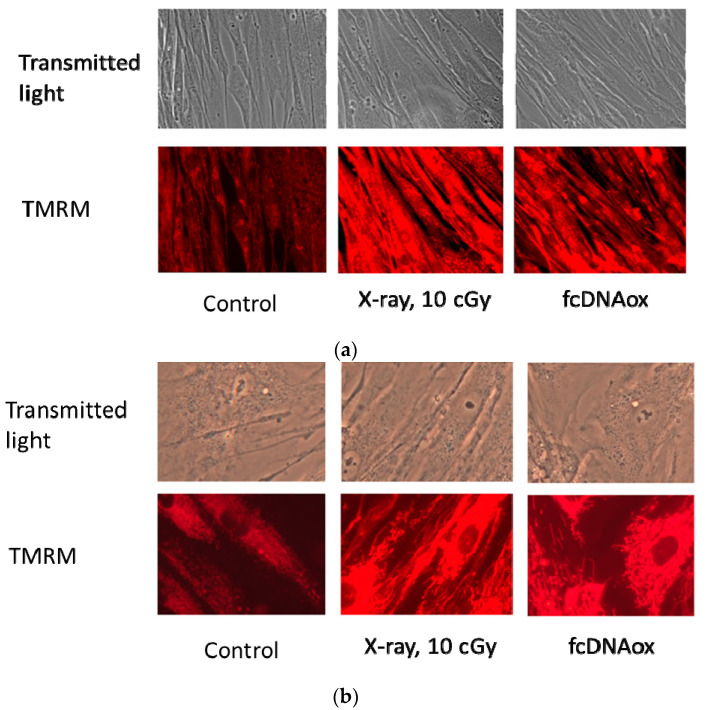
Mitochondrial potential in MSCs one hour after exposure to X-ray radiation (10 cGy) or oxidized cfDNA (50 ng/mL); (**a**) the magnification is 40×, (**b**) the magnification is 100×.

Both radiation (10 cGy) and oxidized cfDNA resulted in an increase in fluorescence level of TMRM after 0.5–3 h by a factor of 2–3. An increase in fluorescence is a result of an increase in the mitochondrial membrane potential, which could be quantitatively assessed using special calibration curves. Based on the previous studies, such calibration plots are linear [24]. Therefore, we can assume an increase in the mitochondrial potential also by a factor of 2–3. Within 24 h, the fluorescence decreased to the control values. Fluorescence microscopy experiments confirm the results obtained by flow cytometry (Figure 2).

### 2.3. Effects of Low-Dose Radiation and Oxidized fcDNA on The Transcriptional Activity of Genes Regulating Mitochondrial Functions

Some researchers demonstrated that radiation-induced oxidative stress affect the expression of genes of the mitochondrial respiratory I–V complexes [14,25].

#### 2.3.1. Mitochondrial Respiratory Complex I

**Figure 3:**
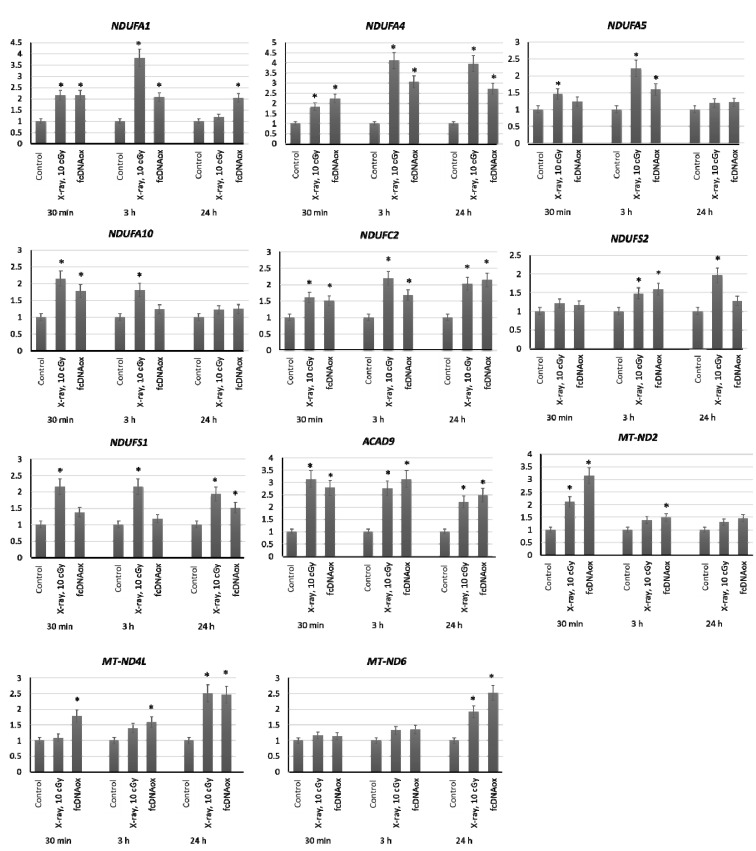
The expression of genes of the mitochondrial respiratory Complex I influenced by low-dose radiation (10 cGy) and oxidized cfDNA (50 ng/mL) assessed by real-time PCR. The genes are indicated in the figure. Ratios to control values are presented. The *TBP* gene was used as an internal standard gene. Mean values were calculated from five independent measurements; (*) denotes a significant difference from control experiments, *p* < 0.001, nonparametric U-test.

We have studied the expression of nuclear DNA genes *NDUFA1, NDUFA4, NDUFA5, NDUFA10, NDUFB10, NDUFC2, NDUFS7, NDUFS2, NDUFV1, NDUFS1, NDUFA9,* and *ACAD9* as well as mitochondrial DNA genes *ND6, ND5,* and *ND4L* that control the synthesis of proteins, which are the part of the Complex I of the mitochondrial respiratory chain or regulate its function. The expression was analyzed after irradiation of MSCs at a dose of 10 cGy and after the addition of oxidized cfDNA fragments to the MSC culture medium (Figure 3).

The results demonstrate that both radiation at a dose of 10 cGy and cfDNAox causes an increase in the expression of nuclear DNA genes *NDUFA1, NDUFA4, NDUFA5, NDUFA10, NDUFC2, NDUFS2, NDUFS1,* and *ACAD9* as well as mitochondrial DNA genes *ND4L, ND2,* and *ND6* within 3 h after exposure. For several genes of the Complex I, the expression was increased for 24 (Figure 3).

#### 2.3.2. Mitochondrial Respiratory Complex II

The expression of *SDHB SDHA,* and *SDHC* nuclear genes that control synthesis of proteins, which are the part of the Complex II of the mitochondrial respiratory chain in MSCs, did not changed within 24 h after irradiation at a dose of 10 cGy and after the addition of oxidized cfDNA to the MSC culture medium.

#### 2.3.3. Mitochondrial Respiratory Complex III

**Figure 4:**
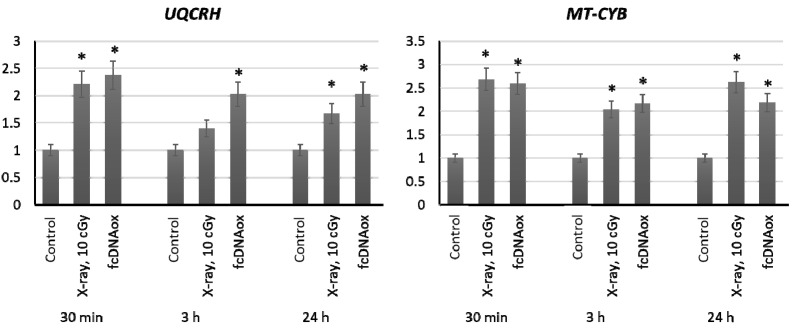
The expression of genes of the mitochondrial respiratory Complex III influenced by low-dose radiation (10 cGy) and oxidized cfDNA (50 ng/mL) assessed by real-time PCR. The genes are indicated in the figure. Ratios to control values are presented. The *TBP* gene was used as an internal standard gene. Mean values were calculated from five independent measurements; (*) denotes a significant difference from control experiments, *p* < 0.001, nonparametric U-test.

We have studied the expression of nuclear DNA genes *BCS1L, CYC1, UQCRFS1*, *UQCRC1*, and *UQCRH* as well as mitochondrial DNA gene *CYB* that control synthesis of proteins, which are the part of the Complex III of the mitochondrial respiratory chain or regulate its function. Irradiation of MSCs at a dose of 10 cGy and the addition of oxidized cfDNA fragments to the MSC culture medium resulted in an increased expression of the nuclear DNA *UQCRH* gene and mitochondrial DNA *CYB* gene, which remains increased for 24 h (Figure 4).

#### 2.3.4. Mitochondrial Respiratory Complex IV

**Figure 5:**
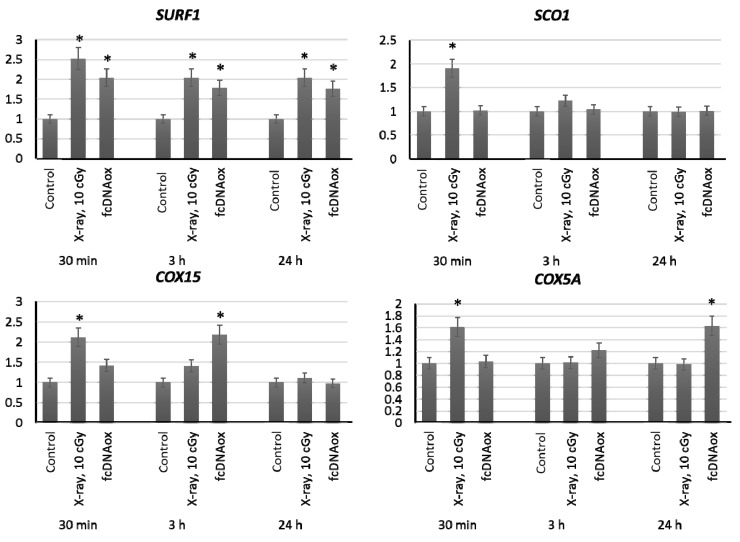
The expression of genes of the mitochondrial respiratory Complex IV influenced by low-dose radiation (10 cGy) and oxidized cfDNA (50 ng/mL) assessed by real-time PCR. The genes are indicated in the figure. Ratios to control values are presented. The *TBP* gene was used as an internal standard gene. Mean values were calculated from five independent measurements; (*) denotes a significant difference from control experiments, *p* < 0.001, nonparametric U-test.

We have studied the expression of nuclear DNA genes *SURF1, COX4, SCO1, COX10, COX15, COX18,* and *COX5A* that control synthesis of proteins, which are the part of the complex IV of the mitochondrial respiratory chain or regulate its function. Irradiation at a dose of 10 cGy or the addition of standard oxidized cfDNA fragments to the MSC culture medium resulted in an increased expression of the *SURF1, SCO1, COX15,* and *COX5A* genes within 0.5–3 h, which remains increased for 24 h (Figure 5).

#### 2.3.5. Mitochondrial Respiratory Complex V

**Figure 6:**
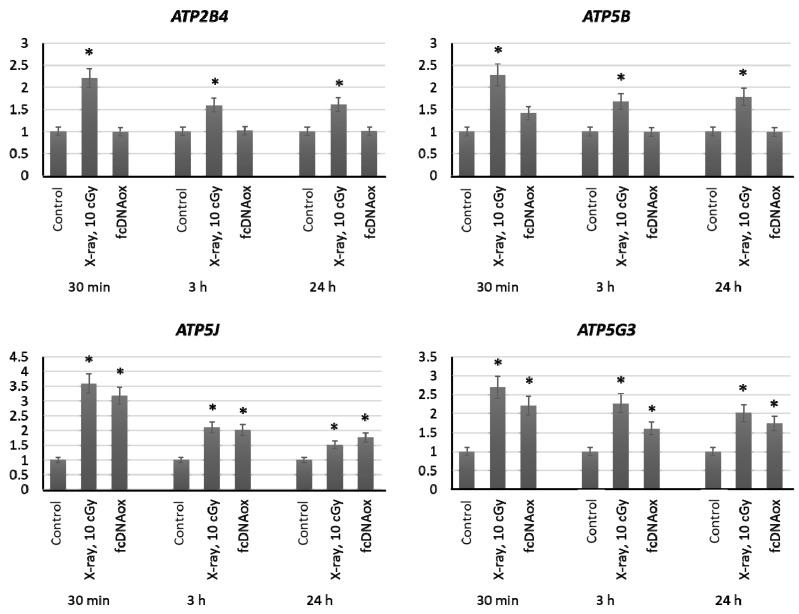
The expression of genes of the mitochondrial respiratory Complex V influenced by low-dose radiation (10 cGy) and oxidized cfDNA (50 ng/mL) assessed by real-time PCR. The genes are indicated in the figure. Ratios to control values are presented. The *TBP* gene was used as an internal standard gene. Mean values were calculated from five independent measurements; (*) denotes a significant difference from control experiments, *p* < 0.001, nonparametric U-test.

We have studied the expression of nuclear DNA genes *ATP2B4, ATP5B, ATP5C1, ATP5J,* and *ATP5G3* as well as *ATP6* and *ATP8* mitochondrial DNA genes that control synthesis of proteins, which are the part of the Complex V of the mitochondrial respiratory chain or regulate its function. Irradiation at a dose of 10 cGy or the addition of oxidized cfDNA fragments to the MSC culture medium resulted in an increased expression of these genes within 3 h, which remains increased for 24 h (Figure 6).

To sum, low-dose radiation (10 cGy) and oxidized cfDNA (50 ng/mL) result in an increased expression of many genes regulating the mitochondrial function, and the expression of several genes remained increased within 24 h after the exposure.

### 2.4. Genes of Mitochondrial Fusion and Fission

**Figure 7:**
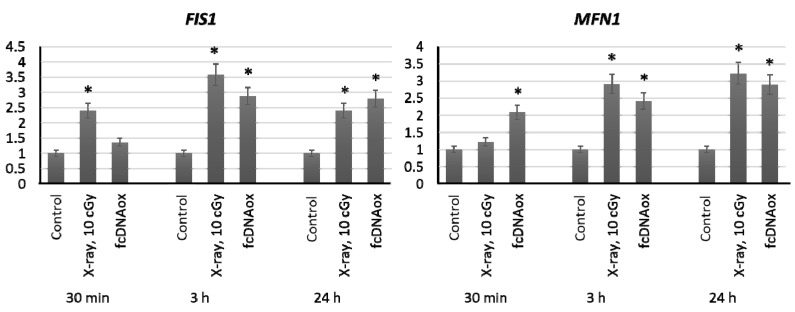
The expression of genes regulating fusion and division of mitochondria influenced by low-dose radiation (10 cGy) and oxidized cfDNA (50 ng/mL) assessed by real-time PCR. The genes are indicated in the figure. Ratios to control values are presented. The *TBP* gene was used as an internal standard gene. Mean values were calculated from five independent measurements; (*) denotes a significant difference from control experiments, *p* < 0.001, nonparametric U-test.

Since mitochondria are highly dynamic organelles that constantly fuse, divide and move, it is useful examine the effect of low-dose of radiation on the expression of genes that regulate mitochondrial fusion and division. FIS1 and DLP1 proteins are involved in mitochondrial division, and MFN1 and MFN2 outer membrane proteins and OPA1 inner membrane protein are involved in fusion. We have shown that low-dose radiation (10 cGy) as well as fcDNAox leads to an increase in expression of *FIS1* and *MFN1* genes by a factor if 2–3 within 3–24 h (*р* < 0.01) (Figure 7).

### 2.5. The Number of Copies of Mitochondrial DNA

**Figure 8:**
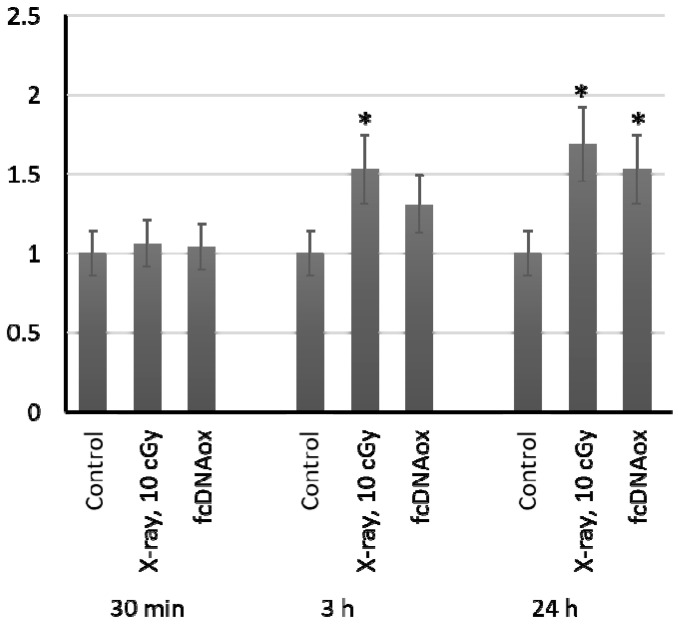
The number of copies of mitochondrial DNA in cells exposed to radiation (10 cGy) or oxidized cfDNA (50 ng/mL) assessed with real-time PCR. Mean values were calculated from seven independent experiments; (*) denotes a significant difference from control experiments, *p* < 0.01, nonparametric U-test.

In 24 h after exposure to radiation at a dose of 10 cGy and oxidized cfDNA, the number of copies of mitochondrial DNA in MSCs increased by 50–65% (Figure 8). This proves the important role of mitochondria in the implementation of the effects of low-dose radiation on MSCs.

### 2.6. Effect of Non-Oxidized DNA Fragments

We used experiments with non-oxidized DNA as a control for the oxidized cfDNA group. The data demonstrate no effect of the addition of non-oxidized DNA on the expression of all studied genes (Table 1).

## 3. Discussion

Let us sum up the main results of our study: (1) X-ray radiation at a dose of 10 cGy as well as oxidized cell-free DNA at a concentration of 50 ng/mL led to an increased expression of a large number of genes regulating the function of the mitochondrial respiratory chain complexes in human mesenchymal stem cells; (2) X-ray radiation at a dose of 10 cGy, as well as cfDNAox, caused a 2–3-fold increase in the expression of the *FIS1* and *MFN1* genes, which regulate the fusion and fission of mitochondria in cells; (3) as a result of LDIR or cfDNAox, the number of copies of mitochondrial DNA in cells increased by 50–65%; (4) low-dose ionizing radiation, as well as oxidized cfDNA, stimulate the formation of quickly repaired DNA breaks in MSCs (these results are presented in Supplementary Materials, Figure S1, Tables S1 and S2). Thus, we can hypothesize that oxidized cell-free DNA may serve as a mediator of MSC response to low doses of X-ray radiation through mitochondria-related genes.

Mitochondria is a major source of reactive oxygen species (ROS). ROS are considered as signaling molecules, beneficial or harmful effects of which depend on their concentration. Low physiological amounts of reactive oxygen species may have positive effects and induce an anti-stress adaptive response [26]. Key signaling pathways controlled by tyrosine phosphorylation such as NF-κB, PKC, MAPK or JNK are activated by primary reactive oxygen species (superoxide anion and hydrogen peroxide) [27]. Current scientific research proves that ROS play an important role in many physiological processes, such as adaptation to hypoxia and physical activity, regulation of autophagy, immunity, differentiation, and longevity [28]. The authors hypothesized that mitohormesis is provided by the formation of reactive oxygen species and a compensatory increase in catalase activity caused by a decrease in glucose availability [29]. This may be an explanation for the concept of mitohormesis—‘low-dose’ mitochondrial ROS may serve as signaling molecules. However, excessive ROS can damage the cell. In the mitochondrial intermembrane space, protein import and folding depend on the formation of disulfide bonds. Therefore, ROS take part in this process [30]. As hydrogen peroxide is a stable product of water radiolysis, occurring at nanomolar concentration upon low-dose ionizing radiation (< 100 mGy) and functioning as the redox master switch in Nrf2/Keap1 and NF-κB/IκB pathways, the authors hypothesize that H_2_O_2_ mediates hormetic effects of low-dose radiation [31].

Here, we have demonstrated that oxidized free-cell DNA can serve as a signaling stress molecule from the family of reactive oxygen species. We have found previously that low-dose X-ray radiation resulted in death of some cells and a consequent increase in oxidized free-cell DNA in the culture medium 21]. Oxidized free-cell DNA is able to penetrate the cytoplasmic membranes and activate genes for key signaling pathways aimed at cell survival [21,22,32]. Moreover, free-cell oxidized DNA may induce the formation of an adaptive response in cells, and the cells become less sensitive to external damaging factors (oxidative stress, high-dose radiation) [21,33,34]. In this case, free-cell DNA acts as a stress signaling molecule [35]. However, the role of mitochondria in the development of the adaptive response of cells to the action of oxidized free-cell DNA has practically not been considered, although the knowledge on the key molecular mechanisms providing the survival of stem cells under irradiation is important in understanding the biology of stem cells.

Proteins of electron transport chain (ETC) are encoded both the genome DNA and mtDNA. Disrupted coordination in expression of these genes resulted in the defected assembly of ETC complexes and, in impaired oxidative phosphorylation and ROS production [36]. A disbalance in this fine-tuned process may lead to the violated import of cytosolic proteins into the mitochondria, the so-called accumulation stress [37]. One solution is to eliminate defective mitochondria through mitophagy [38]. Besides mitophagy, there are other multiple mechanisms that provide a proper mitochondrial function and provide antioxidant defense, compensation mechanisms by the unfolded protein response (UPRmt), and mitochondrial dynamics [39].

Mitochondrial dynamics are the repetitive cycles of fission and fusion of the mitochondrial network. This is an extremely complicated chain of events influenced by many physiological and environmental factors. Prior to mitosis, mitochondria divide to ensure a uniform distribution in daughter cells. The balance between the activity of division and fusion is the key factor determining the structure of the mitochondrial network and its function. Disruption of mitochondrial dynamics can cause various diseases and metabolic imbalances [40,41,42]. Mitochondrial dynamics controls the quality of mitochondria by removing defective organelles and leaving mitochondria with optimal metabolism, intact mtDNA copies and mitochondrial membrane components. This balance between the fusion/fission cycles and mitophagy provides cell homeostasis. If this interplay is disrupted, defective mitochondria remain in the cell, leading to increased ROS levels and mitochondria-induced apoptosis [43]. Before mitophagy, mitochondria are usually fragmented, as this facilitates engulfment by autophagosomes [44]. The fusion of mitochondria provides the diffusion of matrix and membrane components. This serves as a mechanism for the redistribution of proteins and metabolites between healthy and damaged mitochondria. Hence, fusion may recruit defective mitochondria and save them from mitophagy. A balanced interplay of mitochondrial dynamics and mitophagy maintain the quality of both individual mitochondria and the entire mitochondrial network.

Our experiments confirm the activation of mitochondrial dynamics and mitochondrial respiration as a result of LDIR and oxidized free-cell DNA is probably a key mediator of this process. Perhaps, one of mechanisms of radiation hormesis lies in mitohormesis.

## 4. Materials and Methods

### 4.1. Mesenchymal Stem Cells Preparation and Characterizaton

Mesenchymal stem cells were prepared from six adipose tissue samples from the collection of cell cultures of the Research Centre for Medical Genetics (RCMG). Approval #5 was obtained from the Committee for Medical and Health Research Ethics of RCMG (11.12.2019). The samples were mechanically crushed in DMEM medium (Paneco, Moscow, Russia) containing 250 μg/mL gentamicin, 60 U/mL penicillin, and 60 U/mL streptomycin (Paneco, Moscow, Russia). Enzymatic dissociation was performed in DMEM medium through incubating the substance with a 10% fetal calf serum (PAA Laboratories, Vienna, Austria), 0.04% collagenase (Sigma-Aldrich, St. Louis, MO, USA) and the abovementioned antibiotics for 16 h at 37 °C. The cells were centrifuged (200 g, 10 min), transferred into flasks and cultured at 37 °C in AmnioMax C-100 Basal Medium (Gibco Fisher Scientifics, Waltham, MA, USA) with AmnioMax Supplement C-100, 20 μmol/L HEPES (Paneco, Moscow, Russia) and the antibiotics. The study of the expression of surface proteins was carried out by flow cytometry using the relevant antibodies on a CyFlow device (Partec, Meckenheim, Germany). The MSCs were typed by surface antigens: HLA-ABC+, CD44+, CD54 (low), CD90+, CD106+, CD29+, CD49b (low), and CD105 (low).

### 4.2. Irradiation of Cells

The MSCs were irradiated in a growth medium at 20 °C with an ARINA-3 ulsed X-ray radiation unit (Spektroflesh, St.-Petersburg, Russia). The voltage on the X-tube was ∼160 kV, peak energy was 60 keV, and dose rate was 10 cGy/min. 

### 4.3. Preparation of Standardized Oxidized Cell-Free DNA

We prepared model fragments of oxidized DNA to exclude the influence of interfering factors, such as the level of methylation and variations in the content of various sequences. Oxidized DNA samples were prepared by treating genomic DNA (gDNA) samples in the presence of 300 mM H_2_O_2_/Fe^2+^/EDTA or 300 mM H_2_O_2_ (all the reagents from Sigma-Aldrich, St. Louis, MO, USA) with ultraviolet irradiation (λ = 312 nm), which catalyzes degradation of H_2_O_2_ and formation of free radicals (gDNAox). We quantified the oxidation marker (8-oxo-2′-deoxyguanosine, 8-oxo-dG) in the obtained DNA samples by mass spectrometry (ESI-MS) (Q Exactive Quadrupole-Orbitrap Mass Spectrometer, Thermo Fisher, Waltham, MA, USA). The content of 8-oxo-dG was 300–400 per 1,000,000 DNA nucleosides.

### 4.4. Assessment of DNA Breaks with Comet Assay

Single nucleus electrophoresis (Comet assay) with agarose slides was used for the assessment of the number of single- and double-stranded DNA breaks according to the standard protocol [45]. The samples were treated for 2 h with lysis buffer solution [2.5 M NaCl, 0.1 M EDTA (pH 10.0), 10 mM Tris (pH 9.6), 1% N-lauryl sarcosinate, 10% DMSO, 1% Triton X-100] at 25 °C (Sileks, Moscow, Russia). Next, the samples were placed into an electrophoresis chamber in an alkaline buffer [0.3 M NaOH, 1 mM EDTA (pH 10.0)] (Sigma-Aldrich, St. Louis, MO, USA) for 20 min at 15 °C. Electrophoresis was performed at 300 mA for 20 min at 15 °C. Next, the samples were fixed, stained with a 10% ethidium bromide (Paneco, Moscow, Russia) and analyzed with a AxioImagerA2 fluorescence microscope (Carl Zeiss, Oberkochen, Germany) (×40) using the CaspLab software. Two parameters were determined: the tail moment of the DNA comets (MomentT, conventional units) and the percentage of DNA in the tail (DNAtPr, %) per 100–150 cells. Removal of outliers (dying cells, artifacts) from the samples was carried out according to the three-sigma rule. Thus, we did not include cells in which the studied parameter deviated from the mean by more than three standard deviations but detected the percentage such ‘spikes’ in relation to the total number of cells. As the samples were not normally distributed, robust estimates of the mean and SD were used, namely, medians and interquartile ranges divided by 1.349. Free software package PAST version 2.17c was used (the latest version is available at https://past.en.lo4d.com/windows, accessed on 20 December 2021) [46]. The significance level was *p* = 0.05.

### 4.5. Flow Cytometry

Gene expression was assessed by real-time polymerase chain reaction (PCR). RNA was isolated from the cells using YellowSolve kits (Klonogen, St.-Petersburg, Russia) according to the standard procedure, followed by phenol–chloroform extraction and precipitation with chloroform and isoamyl alcohol (49:1). The RNA concentration was determined using the Quant-iT RiboGreen RNA reagent (MoBiTec, Göttingen, Germany) on a plate reader (EnSpire equipment, Turku, Finland), λ_ex_ = 487 nm, λ_fl_ = 524 nm. According to the standard procedure, the reverse transcription reaction was carried out using reagents from Sileks (Moscow, Russia). PCR was performed using the appropriate primers (Synthol, Moscow, Russia) and the SYBR Green PCR Master Mix (Applied Biosystems, Foster City, CA, USA) on a StepOnePlus device (Applied Biosystems, Foster City, CA, USA). The experimental error was approximately 2%. TBP was used as a reference gene.

The primers are listed below as (F; R)

ACAD9 (F:5′-CTCAAGACTAGGGGAGATCATCA-3′; R:5′-ACGCCAGTTTAGGCAAGTATTT-3′);

NDUFA1 (F:5′-ATGTGGTTCGAGATTCTCC-3′; R:5′-GCAACCCTTTTTTCCTTGC-3′);

NDUFA4 (F:5′-CAGAGCCCTGGAACAAACTGGG-3′; R:5′-GACCTTCATTCTAAAGCAGCG-3′);

NDUFA5 (F:5′-GAGAAGCTGGCTATGGTTAAAGCG-3′; R:5′-CCACTAATGGCTCCCATAGTTTCC-3′);

NDUFA9 (F:5′-GTCACGTTCTGCCATTACTGC-3′; R:5′-GGTGGTTGACAACATATCGCC-3′);

NDUFA10 (F:5′-CACCTGCGATTACTGGTTCAG-3′; R:5′-GCAGCTCTCTGAACTGATGTA-3′);

NDUFB10 (F:5′-TAGAGCGGCAGCACGCAAAG-3′; R:5′-CTGACAGGCTTTGAGCCGATC-3′);

NDUFC2 (F:5′-GGTTTGCATCGCCAGCTTC-3′; R:5′-CAGGAAAATCCTCTGGATG-3′);

NDUFS1 (F:5′-AAGCCAGGGAAGGTGTGATG-3′; R:5′-CTGGTCCTGCAGATCACATTCA-3′);

NDUFS2 (F:5′-ACCCAAGCAAAGAAACAGCC-3′; R:5′-AATGAGCTTCTCAGTGCCTC-3′);

NDUFS7 (F:5′-CTTCGCAAGGTCTACGACCAG-3′; R:5′-GGAATAGTGGTAGTAGCCTCCTC-3′);

NDUFV1 (F:5′-TGAGACGGTGCTGATGGACTTC-3′; R:5′-AGGCGGGCGATGGCTTTC-3′);

ND1 (F:5′-CTACTACAACCCTTCGCTGAC-3′; R:5′-GGATTGAGTAAACGGCTAGGC-3′);

ND2 (F:5′-CATATACCAAATCTCTCCCTC-3′; R:5′-GTGCGAGATAGTAGTAGGGTC-3′);

ND4 (F:5′-CTAGGCTCACTAAACATTCTA-3′; R:5′-CCTAGTTTTAAGAGTACTGCG-3′);

ND4L (F:5′-TAGTATATCGCTCACACCTC-3′; R:5′-GTAGTCTAGGCCATATGTG-3′);

ND5 (F:5′-TCGAATAATTCTTCTCACCC-3′; R:5′-TAGTAATGAGAAATCCTGCG-3′);

ND6 (F:5′-CAATAGGATCCTCCCGAATCAAC-3′; R:5′-GTTAGCGATGGAGGTAGGATTG-3′);

SDHB (F:5′-AAATGTGGCCCCATGGTATTG-3′; R:5′-AGAGCCACAGATGCCTTCTCTG-3′);

SDHA (F:5′-TGGTTGTCTTTGGTCGGG-3′; R:5′-GCGTTTGGTTTAATTGGAGGG-3′);

SDHС (F:5′-CTTGCCTACTCTCGGCCTAGAA-3′; R:5′-GACCCTCAGCACAAATCAAAGC-3′);

BCS1L (F:5′-GCCGAACGCAGCTTCCCCAA-3′; R:5′-GGGGTGTTACGAAAACCGCCG-3′);

CYC1 (F:5′-GAGGTGGAGGTTCAAGACGG-3′; R:5′-TAGCTCGCACGATGTAGCTG-3′);

UQCRFS1 (F:5′-GCGTCATAGAACCCAGAAGGAA-3′; R:5′-TGTGGGTCCCTCAACTGTGA-3′);

UQCRC1 (F:5′-AATGGGGCAGGCTACTTTTT-3′; R:5′-GGTCAAGTCTGCACGAGACA-3′);

UQCRH (F:5′-AGGGACCATTGCGTGGCC-3′; R:5′-AGCTACCAGCCTAAGCCAAA-3′);

CYB (F:5′-ATCACTCGAGACGTAAATTATGGCT-3′; R:5′-TGAACTAGGTCTGTCCCAATGTATG-3′);

SURF1 (F:5′-GTGGGGCCTATGTGGTCAC-3′; R:5′-CCTGGGAACGAACCCTCTATTT-3′);

COX4 (F:5′-ATGTCAAGCACCTGTCTGC-3′; R:5′-CCCTGTTCATCTCAGCAAA-3′);

SCO1 (F:5′-GACACATCGGCAAGCCTTTAC-3′; R:5′-ACCCAAGTAGTCCTTGTCAGTT-3′);

COX10 (F:5′-GCAAGTGTATGATTTGCCAGGA-3′; R:5′-TGCAGTGGTACTTACAACCAGA-3′);

COX15 (F:5′-TCACACCGAATGTGGGGTC-3′; R:5′-AGAACACGTCCTTTCATGCCA-3′);

COX18 (F:5′-TCGTGCAAATCAGTTGGGGT-3′; R:5′-GGTGGCAGTTATCTCGCACAT-3′);

COX5A (F:5′-ATCCAGTCAGTTCGCTGCTAT-3′; R:5′-CCAGGCATCTATATCTGGCTTG-3′);

ATP2B4 (F:5′-CGTGGTGTTAGTGACTGCCT-3′; R:5′-GGGAGCTGGATGAGTTGACC-3′);

ATP5B (F:5′-CCCTTCTGCTGTGGGCTATC-3′; R:5′-AAACGTAGTAGCAGGGGCAG-3′);

ATP5C1 (F:5′-TCACCAGGAGACTAAAGTCCATC-3′; R:5′-TATTTTGCTGCCGCTACCATT-3′);

ATP5J (F:5′-GGAGGACCTGTTGATGCTAGT-3′; R:5′-TGGGGTTTTTCGATGACTTCAAA-3′);

ATP5G3 (F:5′-CCAGAGTTGCATACAGACCAAT-3′; R:5′-CCCATTAAATACCGTAGAGCCCT-3′);

ATP6 (F:5′-CACTAAAGGACGAACCTGATCTC-3′; R:5′-GATAGTTGGGTGGTTGGTGTAA-3′);

ATP8 (F:5′-CCGTATGGCCCACCATAAT-3′; R:5′-AGGGAGGTAGGTGGTAGTTT-3′);

FIS1 (F:5′-GATGACATCCGTAAAGGCATCG-3′; R:5′-AGAAGACGTAATCCCGCTGTT-3′);

MFN1 (F:5′-ATGACCTGGTGTTAGTAGACAGT-3′; R:5′-AGACATCAGCATCTAGGCAAAAC-3′);

The method for determining the copy number of mitochondrial DNA is described in the article [33]. Briefly, the PCR reaction mixture (25 μL) consisted of 2.5 μL of a PCR buffer solution (700 mM Tris-HCl, pH 8.6, 166 mM ammonium sulfate, and 35 mM MgCl_2_), 2 μL 1.5 mM deoxynucleotide (dNTP) solution mix; 1 μL 30 pM primer solution, cDNA. PCR conditions were selected for each pair of the primers. The conditions for most primers were as follows: after denaturation (95 °С, 4 min), 40 amplification cycles were carried out for 5 min (94 °С–20 s, (56–62) °С–30 s, 72 °С–30 s). The PCR reaction was carried out in a StepOnePlus device (Applied Biosystems, Foster City, CA, USA). Gene expression was analyzed in several independent experiments using cells from different donors. The results were processed using the software supplied with the instrument.

Real-time PCR data processing methods are based on the equation:

*C*(*T*) = −(1/log*E*)log*P*_0_ + log*PC*(*T*)/log*E*

Real-time PCR data processing is based on plots and comparison of data. The calibration plot method involves the construction of a calibration plot of *C*(*T*) versus log*P*_0_ with a series of dilutions of the reference DNA solution, from which the substrate concentration (*P*_0_) is found in experimental samples. Since we determined only a relative concentration of the substrate, to make a calibration plot, we used a series of dilutions of different experimental samples and compared the efficiencies. The error was 1.2%. All experimental values fell within the linear range of the calibration plot. The reaction efficiency can be calculated as *E* = 10 − (1/*k*), where *k* is a slope of the calibration function *C*(*T*) = klog *P*_0_ + *b*, obtained by linear approximation of the experimental data. At *E* = 2 (or 100% the maximum theoretically possible value) *k* ~ –3.32. In our case, *k* > −3.8, and the efficiency is *E* > 1.82 (or >91%). The PCR conditions (above all, the amplification efficiency) of the series of standards (GAPDH, TBP) are similar to the PCR conditions of the experimental samples. Therefore, we carried out a direct comparison of the expression data of the studied genes using the StepOnePlus software (Applied Biosystems, Foster City, CA, USA). The data obtained using the software and calculated by comparing the concentrations based on the calibration curves were the same. In a series of experiments, good reproducibility of the results was obtained, the error was ~2%.

### 4.6. ROS Visualization with Fluorescence Microscopy

An AxioImagerA2 microscope (Carl Zeiss, Oberkochen, Germany) was used for fluorescent microscopy of cells. The cells were cultured in slide flasks. After irradiation or incubation with cfDNAox, the medium was removed, cells were washed with PBS, and dichloro-dihydro-fluorescein diacetate was added (a stock solution 2 mg/mL was diluted with PBS 1:200 before using). After incubation for 15 min, the cells were washed with PBS and immediately photographed. No less than 100 fields of view were analyzed; fluorescence intensity per cell and the total fluorescence were analyzed using microscope software.

### 4.7. Statistical Analysis

Data were analyzed with Excel, Microsoft Office (Microsoft, Redmond, USA), Statistica 6.0 (Dell Round Rock, Texas, USA), and StatGraphics (Statgraphics Technologies, The Plains, Virginia, USA). The significance of the observed differences was analyzed with the nonparametric Mann–Whitney U-test. The *p*-values < 0.05 were considered statistically significant.

## 5. Conclusions

Oxidized cell-free DNA may serve as a mediator of MSC response to low doses of X-ray radiation through mitochondria-related genes. LDIR results in upregulation of genes controlling respiratory chain, fusion/fission, and increasing the number of copies of mtDNA This effect may be realized through oxidized cfDNA, which plays a role of a stress signaling molecule. These findings indicate the involvement of mitochondria in the response of MSC to low-dose radiation.

## Figures and Tables

**Table 1:** Effects of non-oxidized DNA on the expression of the studied genes in MSC as ratios to control values (mean ± SD, *n* = 5).

Gene	30 min	3 h	24 h	Gene	30 min	3 h	24 h
*NDUFA1*	1.0 ± 0.3	1.1 ± 0.3	1.3 ± 0.3	*UQCRH*	1.0 ± 0.3	1.2 ± 0.3	1.3 ± 0.3
*NDUFA4*	1.0 ± 0.2	1.1± 0.2	1.3 ± 0.2	*MT-CYB*	0.9 ± 0.2	1.1 ± 0.2	1.0 ± 0.2
*NDUFA5*	0.9 ± 0.3	1.0 ± 0.3	1.2 ± 0.3	*UQCRH*	1.1 ± 0.3	1.0 ± 0.3	1.3 ± 0.3
*NDUFA10*	1.0 ± 0.3	0.9 ± 0.4	1.3 ± 0.4	*MT-CYB*	1.2 ± 0.3	1.2 ± 0.3	1.3 ± 0.3
*NDUFC2*	1.2 ± 0.2	1.0 ± 0.2	1.3 ± 0.3	*SURF1*	0.9 ± 0.2	1.0 ± 0.2	1.3 ± 0.3
*NDUFS2*	1.1 ± 0.3	1.1 ± 0.3	1.1 ± 0.3	*SCO1*	1.0 ± 0.3	0.9 ± 0.2	1.2 ± 0.3
*NDUFS1*	1.0 ± 0.3	1.0 ± 0.2	1.3 ± 0.3	*COX15*	1.0 ± 0.3	1.0 ± 0.2	1.3 ± 0.3
*ACAD9*	1.1 ± 0.3	0.9± 0.3	1.2 ± 0.3	*COX5A*	0.9 ± 0.2	1.1 ± 0.2	1.3 ± 0.2
*MT-ND2*	0.9 ± 0.2	1.2 ± 0.3	1.3 ± 0.3	*ATP2B4*	1.0 ± 0.3	1.0 ± 0.2	1.2 ± 0.2
*MT-ND4L*	1.0 ± 0.3	1.1 ± 0.3	1.1 ± 0.3	*ATP5B*	1.1 ± 0.2	1.1 ± 0.3	1.3 ± 0.3
*MT-ND6*	1.1 ± 0.3	1.0 ± 0.2	1.2 ± 0.3	*ATP5J*	0.9± 0.2	1.1 ± 0.2	1.3 ± 0.2
-	-	-	-	*ATP5G3*	1.0 ± 0.3	1.0 ± 0.3	1.1 ± 0.3

## Data Availability

The datasets used and/or analyzed during the current study are available from the corresponding author on reasonable request.

## Supplementary Materials

The following supporting information can be downloaded at: https://www.mdpi.com/article/10.3390/ijms23010261/s1.
