# Supplementary material for: Effect of Low-Dose Ionizing Radiation on the Expression of Mitochondria-Related Genes in Human Mesenchymal Stem Cells
Source: Int J Mol Sci. 2021 Dec 27;23(1):261. doi: 10.3390/ijms23010261 (PMC8745621; doi:10.3390/ijms23010261)
Supplement: Supplementary file 1 [file ijms-23-00261-s001.zip › ijms-1509068-supplementary.pdf]

International Journal of *Molecular Sciences*

Supplementary Materials

Effect of Low-Dose Ionizing Radiation on the Expression of Mitochondria-Related Genes in Human Mesenchymal Stem Cells

Svetlana V. Kostyuk, Elena V. Proskurnina *, Marina S. Konkova, Margarita S. Abramova, Andrey A. Kalianov, Elizaveta S. Ershova, Vera L. Izhevskaya, Sergey I. Kutsev and Natalia N. Veiko

Laboratory of Molecular Biology, Research Centre for Medical Genetics, 1 Moskvorechye St., 115522 Moscow, Russia; svet-vk@ya.ru (S.V.K.); mkonkova@gmail.com (M.S.K.); rimargarii@gmail.com (M.S.A.); googlbubu@gmail.com (A.A.K.); es-ershova@rambler.ru (E.S.E.); izhevskaya@med-gen.ru (V.L.I.); kutsev@mail.ru (S.I.K.); satelit32006@yandex.ru (N.N.V.)

* Correspondence: proskurnina@med-gen.ru

An increased intracellular ROS may lead to DNA damage. Single-cell gel electrophoresis (the comet assay) was developed to examine DNA damage [1]. The total number of single- and double-stranded breaks can be assessed with the alkaline comet assay [1–3]. After lysis, the MSCs were subjected to alkaline electrophoresis in agarose gel stained with a DNA-binding dye (ethidium bromide). The DNA damage (strand breaks) was estimated as the percentage of fluorescent DNA in the comet's tail [1].

Figure 1 shows photographs of comets obtained in the alkaline version of the comet assay after exposure of MSCs to 10 cGy radiation and cfDNAox (50 ng/mL). Intact MSCs were used as controls. Both low-dose irradiation (LDIR) and oxidized cell-free DNA fragments (cfDNAox) caused DNA breaks 15–30 min after exposure. Two hours later the number of DNA breaks decreased to the control level and fall below the control values after 24 h (Figure S1, Table S1). Non-oxidized DNA fragments did not cause DNA breaks in the cells (Table S1).

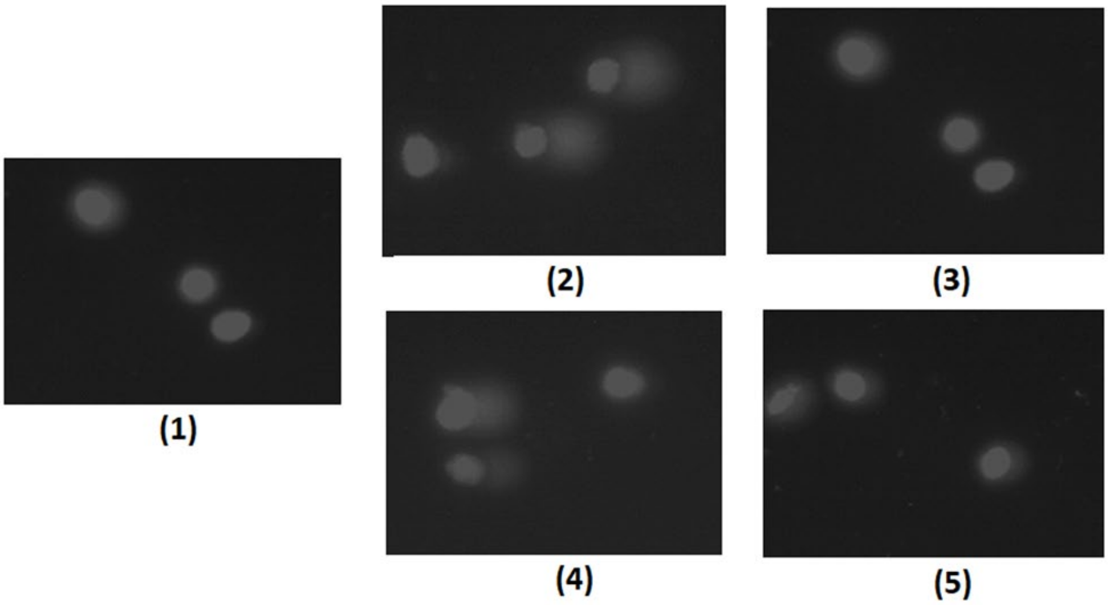

Figure S1. Examples of comets 20 min (**2**,**4**) and 3 h (**3**,**5**) after exposure of MSCs to radiation at a dose of 10 cGy (**2**,**3**) and oxidized cfDNA (50 ng/mL) (**4**,**5**); ethidium bromide staining (40×).

Table S1. The DNA damage after exposure of MSCs to radiation at a dose of 10 cGy (**2,3**) and oxidized cfDNA (50 ng/mL) ($n = 3$); (*) denotes significant difference related to the control cells, $p <$ 0,01 (U-test).

Time	LDIR, 10 cGy	cfDNAox, 50 ng/mL	Non-Oxidized DNA, 50 ng/mL
30 min	2.2 ± 0.5 *	2.4 ± 0.3 *	1.1 ± 0.3
3 h	1.4 ± 0.3	0.9 ± 0.2	1.0 ± 0.2
24 h	0.9 ± 0.2	0.7 ± 0.2	1.1 ± 0.3

We hypothesized that the decrease in the DNA breaks might be a result of the activation of the DNA repair system through signalling cascades regulating DNA repair. BRCA1 and BRCA2 are nuclear proteins involved in the regulation of the cell cycle and the repair of DNA breaks. Both LDIR (10 cGy) and cfDNAox (50 ng/mL) caused a 3.5–4.5-fold increase in the expression of the *BRCA1* and *BRCA2* genes after 30 min (Table S2). The expression of the *BRCA1* genes and *BRCA2* remained 1.7–2.5 times higher than the control values after 3–24 h (Table S2). Thus, LDIR and oxidized fragments of cfDNA increased the number of DNA breaks, but at the same time, DNA repair is activated, which finally leads to a decrease in the number of breaks in cells.

A decrease in the level of breaks and activation of the transcriptional activity of genes that regulate DNA repair leads to a decrease in cell death. Indeed, we have shown that 3 h after irradiation (10 cGy) and exposure to cfDNAox, the transcriptional activity of the *BCL2* antiapoptotic gene increases (Table S2).

Table S2. Expression of the *BCL2* anti-apoptotic gene and *BRCA1* and *BRCA2* repair genes in MSCs exposed to radiation (10 cGy) or cfDNAox (50 ng/mL) assessed with real-time PCR. Mean values were calculated from seven independent experiments; the TBP gene was used as an internal standard gene; (*) denotes significant difference related to the control experiments, $p < 0.001$ (U-test).

Gene	Time	LDIR, 10 cGy	cfDNAox, 50 ng/mL	Non-Oxidized DNA, 50 ng/mL
BCL2	30 min	2.6 ± 0.3 *	2.3 ± 0.2 *	1.1 ± 0.2
	3 h	4.0 ± 0.3 *	3.2 ± 0.3 *	1.0 ± 0.2
	24 h	3.9 ± 0.3 *	2.9 ± 0.2 *	1.3 ± 0.3
BRCA1	30 min	4.6 ± 0.6 *	3.7 ± 0.5 *	1.0 ± 0.2
	3 h	2.5 ± 0.2 *	2.4 ± 0.4 *	1.1 ± 0.3
	24 h	1.9 ± 0.3 *	1.8 ± 0.3 *	1.3 ± 0.2
BRCA2	30 min	3.5 ± 0.6 *	4.2 ± 0.4 *	1.1 ± 0.2
	3 h	2.1 ± 0.3 *	2.6 ± 0.2 *	1.2 ± 0.3
	24 h	1.7 ± 0.2 *	1.7 ± 0.3 *	1.3 ± 0.4

References

1. Lorenzo, Y.; Costa, S.; Collins, A.R.; Azqueta, A. The comet assay, DNA damage, DNA repair and cytotoxicity: Hedgehogs are not always dead. *Mutagenesis* **2013**, *28*, 427–432. https://doi:10.1093/mutage/get018.
2. Collins, A.R. Measuring oxidative damage to DNA and its repair with the comet assay. *Biochim. Biophys Acta* **2014**, *1840*, 794–800. https://doi:10.1016/j.bbagen.2013.04.022.
3. Vasquez, M.Z. Recommendations for safety testing with the in vivo comet assay. *Mutat. Res,* **2012**, *747*, 142–156. https://doi:10.1016/j.mrgentox.2012.05.002.
